# Aptamer-Assisted Detection of the Altered Expression of Estrogen Receptor Alpha in Human Breast Cancer

**DOI:** 10.1371/journal.pone.0153001

**Published:** 2016-04-04

**Authors:** Rajesh Ahirwar, Shamsudheen Karuthedath Vellarikkal, Arghya Sett, Sridhar Sivasubbu, Vinod Scaria, Utpal Bora, Bibhuti Bhusan Borthakur, Amal Chandra Kataki, Jagannath Dev Sharma, Pradip Nahar

**Affiliations:** 1 Department of System and Chemical Biology, CSIR-Institute of Genomics and Integrative Biology, Delhi, India; 2 Academy of Scientific and Innovative research, CSIR- Institute of Genomics and Integrative Biology, Delhi, India; 3 Department of Biosciences and Bioengineering, Indian Institute of Technology, Guwahati, India; 4 Dr. Bhubaneswar Barooah Cancer Institute, Guwahati, Assam, India; University of Wisconsin—Madison, UNITED STATES

## Abstract

An increase in the expression of estrogen receptors (ER) and the expanded population of ER-positive cells are two common phenotypes of breast cancer. Detection of the aberrantly expressed ERα in breast cancer is carried out using ERα-antibodies and radiolabelled ligands to make decisions about cancer treatment and targeted therapy. Capitalizing on the beneficial advantages of aptamer over the conventional antibody or radiolabelled ligand, we have identified a DNA aptamer that selectively binds and facilitates the detection of ERα in human breast cancer tissue sections. The aptamer is identified using the high throughput sequencing assisted SELEX screening. Biophysical characterization confirms the binding and formation of a thermodynamically stable complex between the identified DNA aptamer (ERaptD4) and ERα (*K*a = 1.55±0.298×10^8^ M^-1^; Δ*H* = 4.32×10^4^±801.1 cal/mol; Δ*S* = -108 cal/mol/deg). Interestingly, the specificity measurements suggest that the ERaptD4 internalizes into ERα-positive breast cancer cells in a target-selective manner and localizes specifically in the nuclear region. To harness these characteristics of ERaptD4 for detection of ERα expression in breast cancer samples, we performed the aptamer-assisted histochemical analysis of ERα in tissue samples from breast cancer patients. The results were validated by performing the immunohistochemistry on same samples with an ERα-antibody. We found that the two methods agree strongly in assay output (kappa value = 0.930, p-value <0.05 for strong ERα positive and the ERα negative samples; kappa value = 0.823, p-value <0.05 for the weak/moderate ER+ve samples, n = 20). Further, the aptamer stain the ERα-positive cells in breast tissues without cross-reacting to ERα-deficient fibroblasts, adipocytes, or the inflammatory cells. Our results demonstrate a significant consistency in the aptamer-assisted detection of ERα in strong ERα positive, moderate ERα positive and ERα negative breast cancer tissues. We anticipate that the ERaptD4 aptamer targeting ERα may potentially be used for an efficient grading of ERα expression in cancer tissues.

## Introduction

Breast cancer, a heterogeneous and multifaceted disease of the breast is a major cause of women's mortality around the world [1, 2]. The evidences suggest that the receptors such as the ER and progesterone receptors (PR), which are nuclear proteins, are involved in the carcinogenic growth of breast tissues. The ER are majorly present in the lungs, bones, kidneys, uterus, mammary glands and urogenital tract cells, where they regulate the estrogen-induced proliferation, differentiation and development of vascular and reproductive tissues [3–5]. However, the overexpression and an expansion in the population of breast epithelial expressing ER is linked with the progression and metastasis of breast cancer [5, 6]. Detection of the abnormally expressed receptors for classification of breast tumors into major groups such ER, PR and HER2 positive/negative [7] is an important aspect for making decisions on cancer treatment. A category of ligands called selective estrogen receptor modulators had shown great promises in regressing and inhibiting the growth of ERα-positive cancer [8]. This also established the ERα as a useful target in breast cancer diagnosis and targeted therapeutics [9–11]. Conventionally, the clinical examination of the ERα in breast tissues has been carried out using a radiolabelled ligand binding assay (LBA), enzyme immunoassay (ER-EIA) and immunohistochemistry (IHC) [12–14]. LBA or the dextran-coated charcoal assay (DCC) has been the most primitive method for quantitative detection of ERα in the tissue extracts of breast cancer patients. Although the measures of ERα expression reported by LBA are extremely reproducible [15], the inability of this assay to locate the source of receptor (normal/neoplastic tissue) is a major limitation. Unlike this, the antibody-based immunoassays such as the EIA or IHC provide both qualitative as well as quantitative estimate of ERα in normal and neoplastic tumors [16, 17]. Also, the recent advancement in the mammography and ultrasound techniques has reduced the large sample requirement of LBA and EIA. These made the IHC a preferred method for determination of ERα as it requires lesser sample and provide better description of ERα positive/negative cells in the breast tissue sections.

Currently, the clinical examination of ERα expression in breast tumors is mainly carried out using IHC. Although the IHC assay is easier to perform [18–21], the cases with false positive/negative predictions are increasingly reported these days. This improper management of cancer, which may be attributed to technical flaws and faults in detection methods or the reagent thereof, could pose an additional threat to the lives of cancer patients. As an alternate to antibody in IHC, the use of aptamer for can prove a huge benefit due to low molecular weight of aptamers (better tissue penetration) as well as the high affinity and specificity. Aptamers are a category of short oligonucleotides with an ability to bind targets in a selective manner, and in some cases, inhibit or modulate the activity of targets in a manner similar to conventional therapeutic ligands [22, 23]. They are selected using the method of systematic evolution of ligands by exponential enrichment (SELEX) screening [24, 25]. Aptamer have been tested successfully in analytical applications, as sensing and detection probes, and as therapeutics and drug delivery agents [26–30]. Further, the non-immunogenicity, high stability, better conjugation chemistry, faster and cheaper production without the need for animals make them a molecule of choice for many diagnostic methods [28]. In fact recently, aptamers have been identified against a number of breast cancer marker proteins such as the ERα and HER2 [31–34]. However, many of these studies used conventional procedure wherein an inefficient sequencing method, or oligonucleotide libraries of restricted-diversity [31, 32] have been utilized for aptamer identification. Further, the reported RNA aptamers of ERα [31, 32] appear costly for applications that require the bulk synthesis of the aptamer.

In the present study, we have used a modified SELEX, wherein the entire enriched sequences after nine rounds of ERα-binding were identified with the aid of high throughput Illumina sequencing. We have used a highly diverse aptamer library to find the ERα-binding aptamer and performed scrutinized counter-screenings to exclude the cross-reactive and non-target ERα aptamers. Further, sequences obtained upon high throughput sequencing were sorted by their amplification copy number and top representative sequences were selected as probable ERα aptamers. Analysis of the binding affinity and target specificity was made to narrow down the probable aptamer candidates. One of the DNA sequence is identified as ERα aptamer after measuring its affinity using isothermal titration calorimetry and specificity with flow-cytometry, immunofluorescence, and chromogenic cytochemistry assays. Finally, the clinical utility of the identified aptamer is investigated for its application in the detection of ERα in breast cancer tissue samples.

## Materials and Methods

### Reagents and Materials

Full length recombinant ERα and the ligand binding domain (LBD) of progesterone receptor (PR) were purchased from Thermo Fisher Scientific, USA. The ligand binding domain of ERα and DNA binding domain (DBD) of PR was expressed and purified in our laboratory. Buffer exchange and desalting of proteins were carried out on LMW Zebra spin columns, USA. Cell culture flasks, DMEM, DPBS, Trypsin-EDTA and FBS were purchased from Gibco, Invitrogen, USA. MCF-7 and MDA-MB-231 cells were purchased from ATCC, United States. Trizol, DTT, Protoscript reverse transcriptase, Phusion HF, dNTPs mix, and ethidium bromide were all purchased from Sigma-Aldrich, USA. PVDF was procured from MDI, India. Fluorescein (code: /56FAM/) and biotin (code: /5Biosg/) labelled oligonucleotides (primer/aptamer) were custom synthesized from IDT, USA. All other chemicals used were of analytical grade and purchased from SRL Pvt. Ltd., India. All plastic wares were obtained from Tarsons, India.

### Ethic statement

Surgically resected human breast tissue sections were obtained with written informed consent from patients undergoing breast surgery at Dr. B. Borooah Cancer Institute (BBCI), Guwahati, Assam, India. The study was approved by the Medical Ethics Committee of the Dr. B. Borooah Cancer Institute, Guwahati 781016 in its 15th session (April 8, 2011).

### Expression and purification of ERα-LBD and PR-DBD

Target specific mRNAs that code for ERα-LBD and PR-DBD were obtained by inducing MCF-7 cells with 1 nM β-estradiol (for 26 h) and collecting the total cellular RNA using Trizol method of RNA isolation [35]. First strand cDNA synthesis was performed on the collected RNA using oligo (dT)_20_ primer and Protoscript-II reverse transcriptase (NEB, United States). A specific set of primers for ERα-LBD (forward: 5'-caccacatgagagctgccaacc-3'; reverse: 5'-ctacagggaaaccctctgcctcc-3'), and PR-DBD (forward: 5'-cacctgtttaatctgtggggatgaag-3'; reverse: 5'-ctaaaattttcgacctccaagga-3') were used for second strand cDNA synthesis. The individual cDNAs were then ligation into pET 100/D-TOPO vector and transformed into cloning host TOPO10 cells (Life Technologies, USA). After overnight incubation, the cloned vector was isolated using miniprep plasmid isolation. The respective cloned pET vectors were then transformed into expression host BL21 (DE3) cells. The entire transformation reactions were added to 10 ml of LB broth containing 10 mg/mL ampicillin and grown overnight at 37°C. On the next day, one litre of LB broth containing ampicillin was inoculated separately with overnight grown cultures of the transformed cells carrying ERα-LBD and PR-DBD transcripts, respectively. The culture was grown at 37°C with continuous shaking at 200 RPM till the OD_600_ reached a value of 0.6. Afterwards, the culture was induced with 1 mM IPTG and 1 mM of β-estradiol was added to the broth. Cells were harvested and the expressed proteins (His-tagged) were purified on Ni NTA columns. Additional purification was performed on size exclusion columns (HPLC; HiLoad Superdex 200 PG).

### Synthesis of oligonucleotide library

An oligonucleotide library of 76-nt, comprising a central randomized region of 40-nt with 18-nt flanking sequences at both the ends (5′-ataccagcttattcaatt-N40-agatagtatgtgcaatca-3′) was custom synthesized at 1.3 μmol scale from Integrated DNA Technologies, USA. The obtained yield of the synthesized library was 286.5 nmoles. Primers were also custom synthesized at 0.2 μmol scale. Synthesized sequences and oligonucleotide library were analysed for purity on 15% urea-PAGE prior to their use in SELEX screening.

### Preparation of ERα coated polypropylene beads for SELEX Screening

Target-immobilized solid support for SELEX screening was prepared by immobilizing the full-length ERα (ESR1, Thermo Fisher Scientific) onto polypropylene bead of average diameter 1 mm, which were pre-activated by 1-fluoro 2-nitro 4-azidobenzene (FNAB) linker [36]. The ERα coated polypropylene beads (PP-ERα) were equilibrated in phosphate buffer (10 mM sodium phosphate, pH 7.4) and stored at 4°C until used in SELEX screening.

### *In vitro* Selection of ERα Aptamers using SELEX Screening

In vitro selection of ERα binding DNA aptamers was carried out in a 1.5 mL eppendorf tube. The DNA library (5'- ataccagcttattcaatt -40nt- agatagtatgtgcaatca-3') for the initial round of SELEX screening was prepared by heating 50 nmoles of synthesized library in aptamer binding buffer (20 mM Tris-HCl, pH 7.6, 120 mM NaCl, 5 mM KCl, 1 mM MgCl_2_ and 1 mM CaCl_2_) at 95°C for 10 min followed by rapid cooling on ice. In the first round of SELEX screening, the prepared 50 nmoles DNA library was mixed with 100 pmoles of PP-ERα in total volume of 500 μL and incubated at room temperature for 2 hour. Thereafter, the unbound sequences were removed from the PP-ERα beads by collecting the supernatant solution. The PP-ERα beads were washed thrice with an equal volume (500 μL) of aptamer washing buffer (0.1% Tween-20 supplemented aptamer binding buffer) to remove the non-specifically adhered DNA sequences. The PP-ERα beads retained sequences were then collected using heat elution at 95°C for 10 min in a minimal volume (ca. 300 μL) of the aptamer binding buffer. The collected sequences were PCR amplified using unlabelled forward and biotinylated reverse primers, using the thermal cycling of 95°C- 5 min hot start, 15 cycles of 95°C- 30 s, 46°C- 30 s, 72°C- 30 s, 72°C- 3 min. The amplified duplex sequences were converted to single strands through alkaline denaturation on magnetic streptavidin beads using 150 mM NaOH [37]. Next, a predetermined amount of the enriched single strand library was mixed with new PP-ERα beads and the next round of SELEX screening was initiated. The stringency of selection was changed during each round of selection by varying the amount of interacting ERα and enriched aptamer as shown in S1 Table. Particularly, the mole ratio of ERα to aptamer was varied using a controlled number of ERα-PP beads. Also, two negative SELEX-screenings, each after 4^th^ and 6^th^ round of SELEX enrichment were performed on PP- FNAB beads (without ERα) to remove the matrix binding aptamers. Additionally, a single round of counter-SELEX screening against nuclear extract collected from MDA-MB-231 cells (deficient in ERα) was performed to exclude the DNA sequences having co-affinity for molecules other than ERα. All the screenings were performed at RT (ca. 27°C). Total nine rounds of positive SELEX screenings and two rounds of negative screening and a single round of counter-screening were performed to obtain an enriched pool of sequences having binding affinity and specificity for ERα.

### Monitoring of the SELEX enrichment

Cumulative enrichment of the ERα-binding sequences during the course of SELEX screening was monitored using a modified aptamer-assisted ELISA [37, 38]. Briefly, 250 ng ERα was immobilized to the FNAB-activated wells of a polypropylene microtest plate (APPμTP) by 2 h incubation at 37°C. The collected pools of enriched sequences from various rounds of SELEX screening (round 2, 4, 6, 8 and 9) were biotinylated (via PCR with biotinylated forward primer) and strand separated on magnetic streptavidin beads (Dynabeads® M-280 Streptavidin; Thermo Fisher Scientific) to obtain single stranded sense sequences. These biotinylated sequences were then added to the ERα-immobilized wells and incubation for 1 h at room temperature. After stipulated time, the wells were washed with aptamer binding buffer and then filled with 10 μL of streptavidin-HRP conjugate solution (1:1000 dilution from 1mg/mL). After 1 h of incubation at RT, the unbound conjugate was removed by washing with aptamer washing buffer. Next, the wells were filled with 10 μL of HRP substrate dye (6 mg OPD and 8 μL H_2_O_2_ in 6 mL 0.1 M citrate buffer, pH 5.2) and incubated for 5–10 min to allow the colour development reaction. The reaction was stopped by adding 2 μL of 5% H_2_SO_4_ and the image of the developed colour was captured and quantified using colour saturation parameter [38]. In control, an aptamer-ELISA with biotinylated non-enriched aptamer library was performed. The measured colour values were plotted against individual screening rounds to monitor the enrichment of ERα binding sequences.

### High throughput sequencing

The entire enriched pool obtained at the end of 9^th^ round of SELEX screening (R9) was PCR amplified using unlabelled primers and the correct-size sequences (76-mer) were selected following a gel extraction using a QIAquik kit (QIAGEN Inc. USA) and size separation on 2% agarose gel. Thereafter, 1μg of purified R9 product was subjected to end repair, adenosine (A) base addition and ligation of sequencing adapter using truseq DNA sample preparation kit (Illumina Inc. SAD. USA). Sequencing data was generated on Hiseq2500 platform with 101×2 paired end V3 SBS sequencing chemistry using default parameters. Raw reads were filtered for Phred quality score 30 and sequencing adapter contamination using Trimmomatic PE module [39]. Paired end reads were overlapped and screened for the presence of forward and reverse priming sequence and correct size (76 bp). Subsequently, the priming sequences were removed using seqtk (https://github.com/lh3/seqtk) prior to copy number estimation. Copy numbers of each sequence were estimated and tabulated using 'awk' command line. Fold enrichment of each sequence was calculated as reported elsewhere [40].

### Measuring the relative affinity and specificity of the probable ERα aptamers

A comparison of the relative binding among the selected probable ERα aptamers was made using the aptamer-assisted ELISA [37]. Briefly, the wells of 96-well microplate were coated with 0.5 μg of ERα by overnight incubation at 4°C or 2 h incubation at 37°C. Next, the wells were blocked with 2% BSA and then loaded with 100 μL of 500 nM biotinylated aptamers ERaptD1—ERaptD10 (Table 1). After 2 h of binding at RT, the wells were washed to remove the unbounded sequences and then filled with 100 μL streptavidin-peroxidase conjugate (1:2000 dilution from 1mg/mL). After 30–45 min incubation at RT, the wells were washed with adequate washing buffer to remove excess and unbound conjugate and the bound aptamers were detected colorimetrically. In parallel, a positive control assay (ELISA) with anti-ERα antibody (sc-543, Santa Cruz Biotechnology) was also performed. In another assay, the biotinylated non-enriched aptamer library was used as negative control. Absorbance values corresponding to aptamer binding were normalized against the anti-ERα binding and one-way ANOVA with Tukey's multiple comparison test was performed using GraphPad Prism version 5.03 for Windows, GraphPad Software (San Diego California USA).

Similarly, the target specificity of these probable aptamers was measured on complex sample of ERα using the aptamer-assisted ELISA. Cytoplasmic and the nuclear extracts of MCF-7 and MDA-MB-231 were coated to microtiter plates and incubated separately with each of the probable aptamers. Binding and detection reaction were carried out in a similar way. The anti-ERα antibody and non-enriched aptamer library were used as positive and negative controls, respectively.

**Table 1:** List of the selected most probable ERα aptamers and their copy number as obtained after high throughput sequencing.

Aptamer	Aptamer sequence	Copy no.
ERaptD1	ATACCAGCTTATTCAATTAGGTACAGAAGGCGCGACAGAAACTGCGGTCCCAGGGCGTAGATAGTATGTGCAATCA	17619
ERaptD2	ATACCAGCTTATTCAATTGGCGGGCAGCGATTGTACCGGTACCACCTGGCAATGTAGAAGATAGTATGTGCAATCA	13762
ERaptD3	ATACCAGCTTATTCAATTGGGGATAACGTCGTCACGTCGTGTCATCATTGGTTCAGTCAGATAGTATGTGCAATCA	11998
ERaptD4	ATACCAGCTTATTCAATTCGTTGCATTTAGGTGCATTACGGGGGTTATCCGCTCTCTCAGATAGTATGTGCAATCA	11034
ERaptD5	ATACCAGCTTATTCAATTGCAGCAGTGTCATATGAGGGCGTTCGTCAAATGTGCAGGGAGATAGTATGTGCAATCA	7263
ERaptD6	ATACCAGCTTATTCAATTCGCATAGGCAAAACGGTGCGGTGCATATTCGTGACAAGCGAGATAGTATGTGCAATCA	2829
ERaptD7	ATACCAGCTTATTCAATTGGACGGATGCACACACTACTGACCGGTTTTCGCCAGCCCCAGATAGTATGTGCAATCA	2628
ERaptD8	ATACCAGCTTATTCAATTCGAGTAACGCTGTCTCTTCCGAATCGGGGGAAGGCGGAGGAGATAGTATGTGCAATCA	2406
ERaptD9	ATACCAGCTTATTCAATTCACAGGGCTGTTTTTACGCAATGCTAGTGTTCGACTCAGTAGATAGTATGTGCAATCA	829
ERaptD10	ATACCAGCTTATTCAATTCACTAATACTAGGCGGTCCACGCGCAGTTAAACCGGTCAAAGATAGTATGTGCAATCA	421

### Isothermal titration calorimetry

Thermodynamic parameters of ERaptD4-ERα binding was studied by isothermal titration calorimetry (ITC). All the calorimetry experiments were performed at 25°C using a MicroCal VP-ITC (MicroCal, Inc., Northampton, MA, USA). ERα-LBD and aptamer were reconstituted in 10 mM Tris-HCl buffer (pH 8.0). The sample cell holder contained 300μL of 1.0 μM ERα-LBD and the syringe was filled with 250 μL of 10 μM ERaptD4. The thermal equilibration step at 25°C was followed by an initial 120 s delay step and subsequent 25 injections of 5 μL (3 μL for initial injection) at a spacing of 180 s and continuous stirring of 307 RPM. Each injection generated a heat-burst curve between DP (μcal/sec) versus time (min). The area under each peak was determined by integration, using Origin 7.0 analysis tool to give the measure of heat associated with the injection. The binding affinity and thermodynamic parameters of the binding process were obtained by fitting the integrated heats of binding the isotherm to the one site binding model. A control ITC experiment with the non-enriched aptamer library was performed to ascertain the specificity of binding.

### Competition binding assay

The competition binding assay was performed between ERaptR4 and estrogen response element sequences (5'- ttccagtgccaccggtg -3' and 5'- ggtccagtgtcactggac- 3') to analyse their binding at the DNA binding domain of ERα. Double stranded EREs were prepared following the heat denaturation of each ERE with its complementary sequences and a rapid cooling on ice for 1 hour. The competition assay was performed by first immobilizing the recombinant full length ERα (2μg/well) to the FNAB activated wells of a microtiter plate by 1 h incubation at 40°C. This followed the addition of biotinylated ERaptR4 (100 nM) and an incubation of 2 h at RT. Thereafter, the unbound biotinylated ERaptD4 was removed and the wells were filled with 100 μL of 0–5000 nM of respective EREs. After 2 h of binding at RT, the unbound EREs were removed and the ERα-bound ERaptD4 (biotinylated) was detected by incubating the wells with streptavidin-HRP conjugate and its substrate dye, as described previously. The binding (absorbance) values were normalized from 100% (no ERE) to 0% (non-specific binding in absence of biotinylated ERaptD4) and plotted to an XY graph in GraphPad Prism version 5.03 for Windows.

### Flow cytometry and fluorescence microscopy

Target selectivity of ERaptD4 was determined using flow cytometry and fluorescent microscopy assays. The MCF-7 and MDA-MB-231 cells were prepared for flow cytometry by incubating in a 1% solution of sodium azide for 30 minutes. Thereafter, the cells (~2 ×10^5^) were incubated with 100 nM of fluorescent-labelled (FAM) ERaptD4. After 30 min of binding, the cells were washed with DPBS and fixed in 2% p-formaldehyde. Fluorescence intensity in the treated cells was then recorded in a FACS cytometer (Becton Dickinson Immunocytometry Systems) by counting 10,000 events.

Validation of FACS result was performed using fluorescent microscopy. For this, monolayer cultures of MCF-7 and MDA-MB-231 cells (60–70% confluent) were grown on 0.1% gelatine coated cover slips in serum supplemented DMEM. About an hour before the aptamer treatment, the media was changed to serum-free, phenol red-free DMEM and 500 nM of fluorescent labelled ERaptD4 was added to the cells. After 1 h of incubation, the cells were removed from medium and washed with cold DPBS. Thereafter, the cells were fixed in 2% p-formaldehyde and counterstained with DAPI dye. Finally, the cells were visualized under fluorescence microscope (Advance Fluorescence, Leica Microsystems, India) and images were taken.

### Aptacytochemistry

Aptamer-assisted cytochemistry was performed using biotinylated ERaptD4 on p-formaldehyde fixed monolayer cultures of MCF-7 and MDA-MB-231. Cells were permeabilized by 10 min incubation in 0.1% Triton X-100. Blocking was performed with a mixture of 1% BSA and 22.5 mg/ml glycine in 1X PBS. The primary binding reaction was carried out with 200 nM biotinylated ERaptD4 through 2 h incubation at RT. The secondary binding reaction was performed with 1:500 dilution of streptavidin-HRP conjugate. Afterwards, cover slips were stained with DAB solution (500 μL 1% DAB in 5 mL 1X PBS, 15 μL H_2_O_2_) and after counterstaining with haematoxylin, the cells were visualized under microscope (Nikon Eclipse i9).

### Aptahistochemistry

The formalin preserved paraffin embedded (FPPE) tissue sections of breast cancer patients were baked overnight at 56°C. Deparaffinization was carried out by two washes of xylene for 10 min each. Tissue sections were rehydrated by a sequential wash in 100%, 90%, 70%, 50% ethanol and running tap water for 5 min each. Antigen retrieval was performed in the microwave oven by following two cycles of heating- (i) 850 W for 5 min in Tris-EDTA, pH 9.0; reheating at 850 W for 5 min in deionised water, (ii) heating at 600W for 5 min in Tris-EDATA; reheating at 600W for 5 min in deionised water. Blocking was performed with 1.5% FBS+1% BSA in PBST, pH 7.2 at RT for 1 h. Endogenous peroxidase activity of tissues was blocked with 0.3% H_2_O_2_. The primary binding reaction was performed with 1 μM biotinylated ERaptD4 by overnight incubation at 4°C. The secondary binding reaction was carried out with streptavidin-HRP conjugate (1:500 dilution) for 1 h. Staining was done using DAB staining solution (500 μL 1% DAB in 5 mL 1X PBS, 15 μL H_2_O_2_). Counterstaining was performed with haematoxylin and the images were taken under Nikon Eclipse i9 microscope. In control, the same samples were analyzed using anti-ERα antibody in an IHC assay. A comparison of immunohistochemistry to aptahistochemistry was made by kappa analysis in SPSS software package. The kappa statistics was performed on the labelling indexes (percentage of DAB-stained nuclear area over total nuclear area) obtained from ImmunoRatio analyses (http://153.1.200.58:8080/immunoratio/) [41].

## Results

### High throughput sequencing-assisted selection of ERα binding DNA aptamer

**Fig 1:**
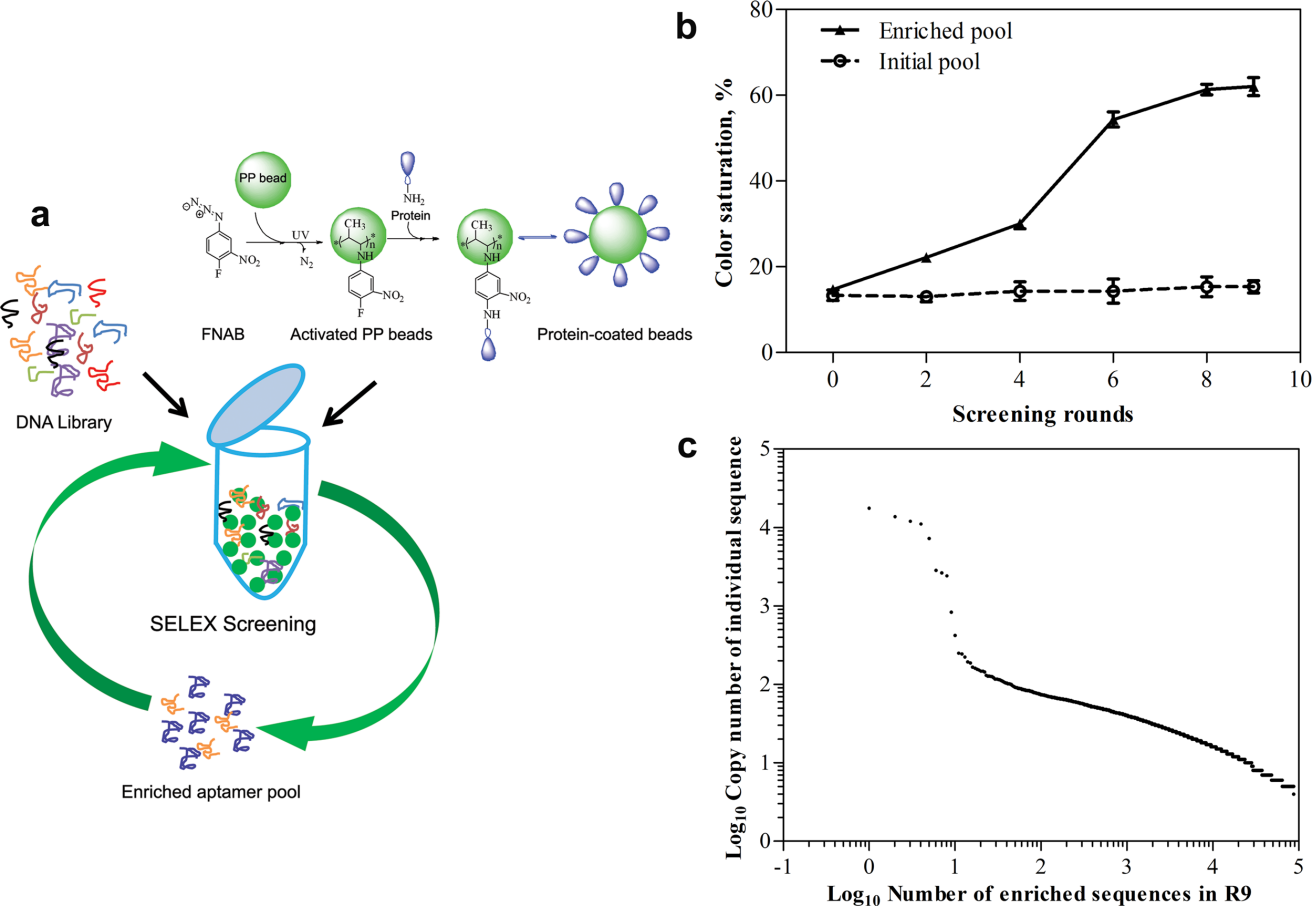
Monitoring the aptamer enrichment. (**a**) Schematic representation of ERα aptamer screening using modified SELEX method. (**b**) Monitoring the enrichment of ERα binding sequences through photocolorimetric approach. The relative binding of enriched sequences (equivalent amounts) obtained after 2, 4, 6, 8 and 9^th^ round of SELEX screening is analyzed through photocolorimetric method. The initial non-enriched library is used as a control sequence. (**c**) Copy number analysis (cut-off = 5) in the enriched DNA sequences obtained through Illumina sequencing.

Selection of ERα binding aptamers was performed using a modified-SELEX on polypropylene bead-coated ERα (PP- ERα) with a negative selection on activated PP beads (Fig 1A). Further, cross reactive aptamers were removed by counter screening of the enriched pool against nuclear extract collected from ERα-deficient MDA-MB-231 breast cancer cells. The total amount of aptamer sequences and the protein (PP- ERα) during various rounds of SELEX screenings was maintained stringently to favor maximum sequence diversity during initial selections and the selective enrichment in later rounds of screening (S1 Table). Enrichment of target-selective sequences during SELEX screening was monitored using an adopted enzyme-linked aptamer assay or the aptamer-ELISA [37]. As shown in Fig 1B, the SELEX pool was found to saturate with high affinity ERα binders by the ninth round of screenings; henceforth, the entire enriched pool was sequenced using high throughput Illumina sequencing. Upon sequencing, we obtained over 3.14 million paired end 101 bp fastq raw reads, which after filtering to remove low quality bases, sequencing adaptors and sequences missing the correct priming sites has remained to 3.03 million reads. As SELEX screening favors logarithmic enrichment of target-binding sequences, we assumed that the DNA sequences having a highest copy number may actually represent the most probable aptamers of ERα. To probe this assumption, we estimated the copy numbers of each sequence and found that only a few sequences in the entire enriched pool were present as high copy number sequences (S2 and S3 Tables). Further, when these sequences were plotted against their individual copy number, we found some of these high copy number sequences located distinctly from the remaining average or low-copy number sequences (Fig 1C). We selected these high copy number sequences as probable ERα aptamers (Table 1) and analyzed their ERα binding affinity *in vitro*.

### Identification of potential ERα aptamer in the enriched ERα-binding DNA pool

**Fig 2:**
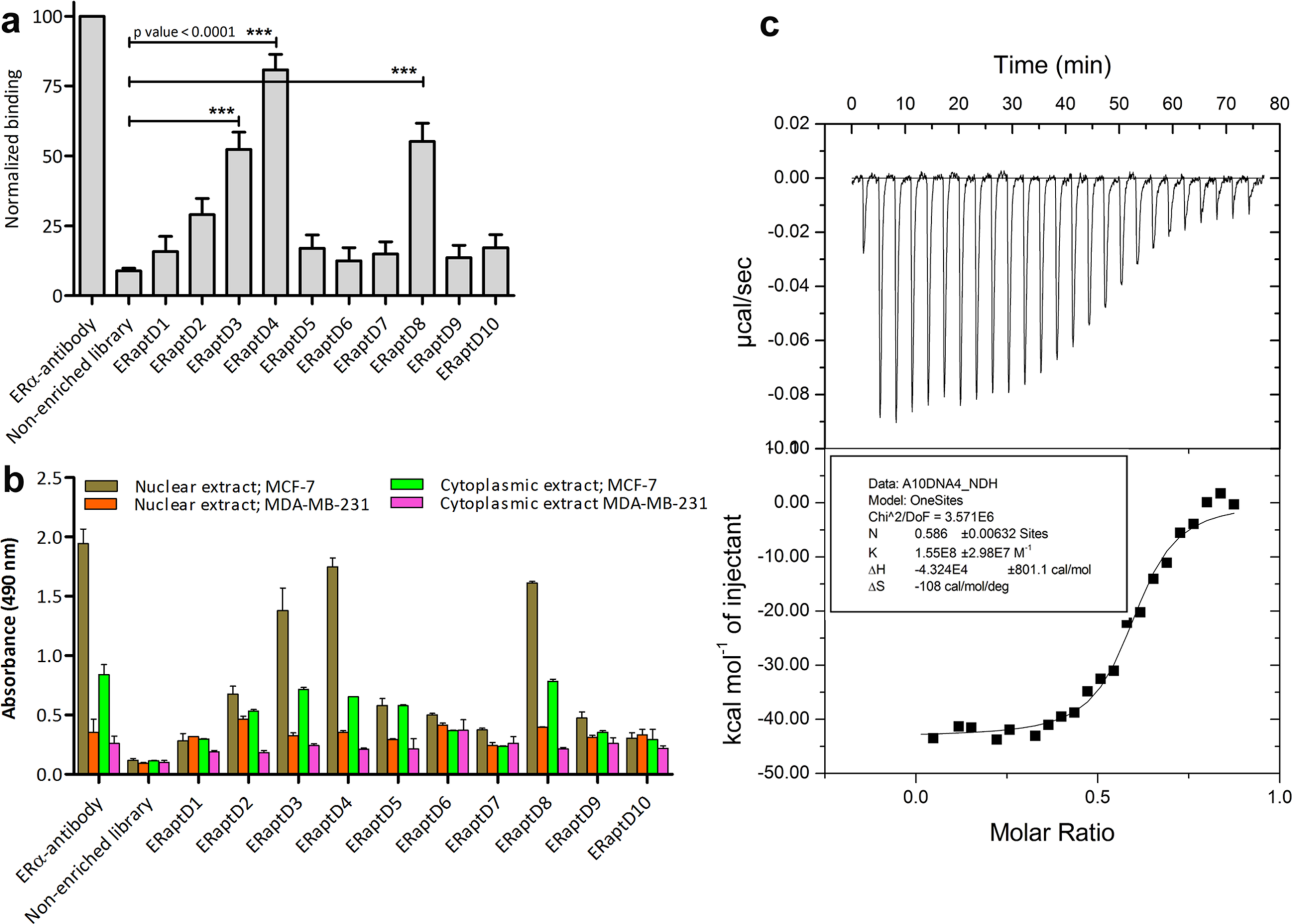
Analysis of the affinity and specificity of selected probable ERα-aptamers. (**a**) Relative binding of selected DNA sequences is analyzed through an aptamer-assisted ELISA. ERα-antibody is used as a positive control to normalize the binding. Non-enriched aptamer library is taken as negative control. (**b**) Target selectivity of selected sequences is analyzed in a similar manner using cellular extracts of MCF-7 and MDA-MB-231 cells. (**c**) ITC isotherms of ERα interactions with aptamer ERaptD4 is determined by titrating aptamer (10 μM; in the syringe) into ERα (1μM, 1.4 ml in sample cell). The top panel represents the raw heats of binding obtained upon titration of aptamer to ERα protein. The lower panel is the binding isotherm fitted to the raw data using one site model.

To evaluate the binding strength between selected sequences (probable aptamers) and ERα, we performed an enzyme-linked aptamer assay wherein the immobilized ERα (0.5 μg/well) in the wells of microtiter plate was titrated against 500 nM each of selected aptamers and the binding was quantified using streptavidin-HRP conjugate and its substrate solution. As evident in Fig 2A, three sequences, namely the ERaptD3, ERaptD4 and ERaptD8 showed a 47.3%, 78.5% and 50.5% relative binding with ERα, respectively. Variability analysis of the obtained absorbance units of ERaptD4 (mean binding = 1.69, +3SD = 2.43, -3SD = 0.97, n = 5), ERaptD8 (mean binding = 1.16, +3SD = 2.07, -3SD = 0.25, n = 5) and ERaptD3 (mean binding = 1.09, +3SD = 1.93, -3SD = 0.27, n = 5) also showed a significant difference from the obtained absorbance units of library control (mean binding = 0.18, +3SD = 0.31, -3SD = 0.06, n = 5). The relative high strength of binding between ERα and these sequences shows their likelihood to emerge as potential ERα aptamers. The rest of the sequences which depicted an insignificant (<10% relative binding) binding could be attributed to the over representation of DNA sequences in initial library or the bias during amplification [42].

Further, as the selected probable aptamers may vary in their target-specificities, we measured their binding against the nuclear and cytoplasmic extracts of MCF-7 and MDA MBA-231 breast cancer cells (Fig 2B). Inclusion of antibody (positive control) and non-enriched library (negative control) was mandated to evaluate the relative target-specificity. Interestingly, the same three sequences, namely ERaptD3, ERaptD4 and ERaptD8 came up with a pattern of relative binding with MCF-7 extract as obtained with the purified ERα protein (Fig 2A and 2B). This suggests that irrespective of the presence of myriad cellular components in the heterogeneous extracts of MCF-7, these three DNA sequences bound selectively to the ERα present in the crude extract. Further, the negligible binding of the same DNA sequences with the cell extracts of ERα-deficient MDA-MB-231 proves the target specificity of these sequences. Based on these observations, we assumed that these sequences can act as potential aptamers to ERα. However, we have only selected ERaptD4 as an aptamer candidate to ERα in the present study and performed reverent experiments to analyze its diagnostic applicability.

Subsequently, we analyzed the thermodynamics of ERaptD4-ERα interaction using ultrasensitive isothermal titration calorimetry assay. As shown in Fig 2C, the thermodynamic parameters suggests that the ERaptD4 aptamer undergoes an enthalpy-driven conformational change upon its binding to the ERα, to give an association constant (*K*a) of 1.55±0.29 ×10^8^ M^-1^ and the binding enthalpy (Δ*H*) and entropy (Δ*S*) of 4.32×10^4^±801.1 cal/mol and -108 cal/mol/deg, respectively. These values are indicative of the strong binding of ERaptD4 aptamer with ERα protein. Further, the lack of binding with non-enriched initial library (S1 Fig) suggests the usefulness of SELEX screening in identifying ERα-specific aptamer.

**Fig 3:**
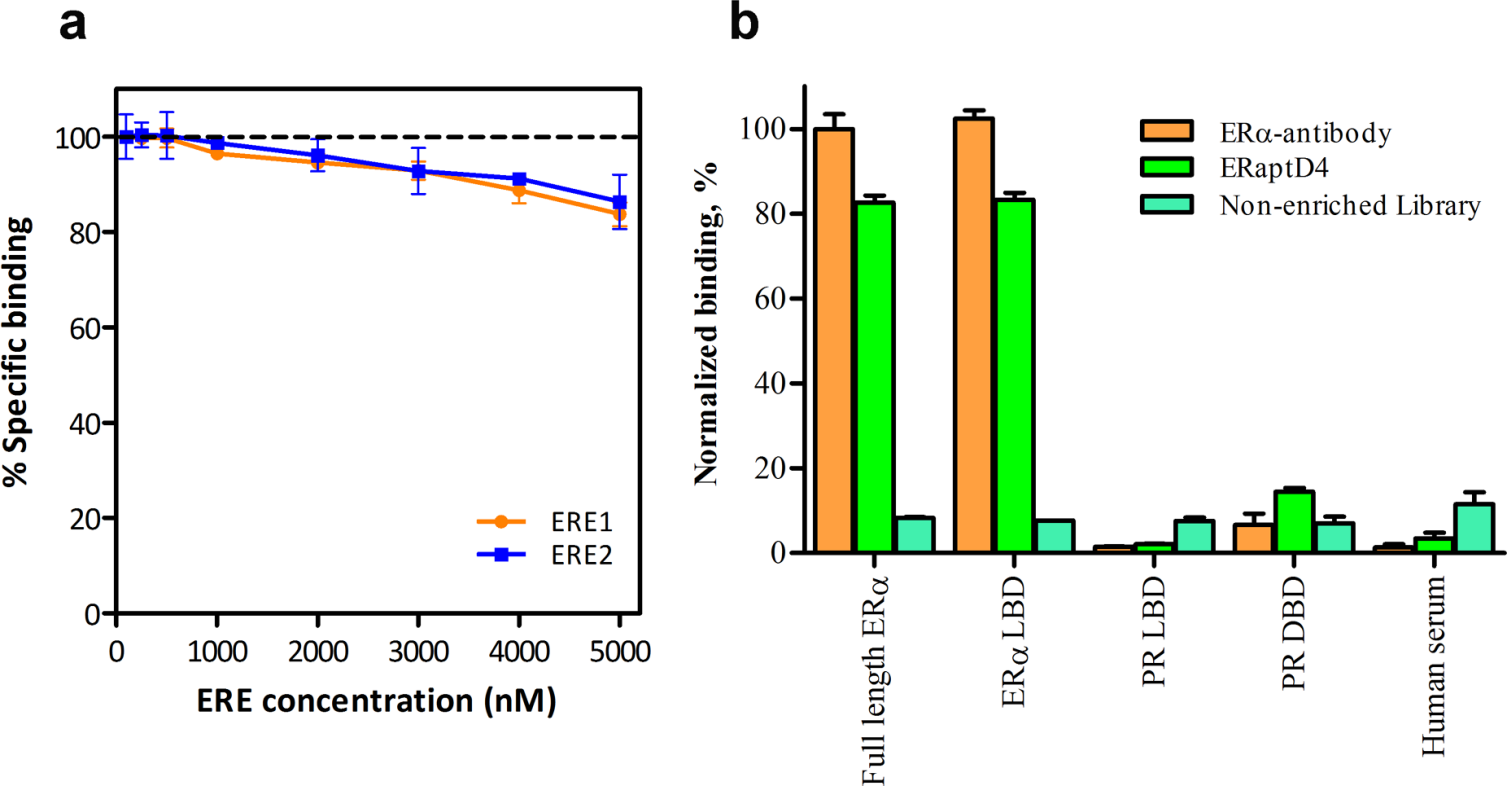
Analysing the binding site of ERaptD4 on ERα. **(a)** Effect of increasing concentrations (100 nM, 250 nM, 500 nM, 1000 nM, 2000 nM, 3000 nM, 4000 nM and 5000 nM) of unlabelled EREs (ERE1: 5'- ttccagtgccaccggtg -3'; ERE2: 5'- ggtccagtgtcactggac- 3') on saturation binding of biotinylated ERaptD4 (100 nM). **(b)** Binding of ERaptD4 to the ligand binding domain and full length ERα is probed using aptamer-ELISA. PR-DBD, PR-LBD and human serum are taken as sample controls. Non-enriched aptamer library is taken as ligand control.

A further insight into the interacting domains of aptamer and ERα was made by competition binding assay and target-selective ELISA. To determine the target domain on ERα (LBD/DBD) to which the ERaptD4 bind, we performed a competition binding assay between biotinylated ERaptD4, whose binding site on ERα was unknown and estrogen response elements, which bind selectively to DNA binding domain of ERα. As shown in Fig 3A, we observed an insignificant reduction of 5% (4.653 ± 3.300) in the binding of ERaptD4 at 20 fold concentration of EREs. This reached a maximum reduction value of 15% (14.957 ± 2.651) at 50-fold concentration of EREs. This lack of competition between two ligands (aptamer and ERE) signifies that the ERaptD4 does not bind the DNA binding domain of ERα. This also suggested that may be the ERaptD4 target LBD for its binding. To confirm this, we performed an aptamer-ELISA against the full length ERα and ERα-LBD (Fig 3B). Surprisingly, the aptamer showed an equivalent binding to both ERα variants (LBD and full length ERα), while a negligible binding was observed for the control proteins like the PR-DBD, PR-LBD and the human serum. These observations showed that the ERaptD4 bind the ligand binding domain of ERα.

### Specific staining of ERα-positive breast cancer cells by the ERaptD4 aptamer

**Fig 4:**
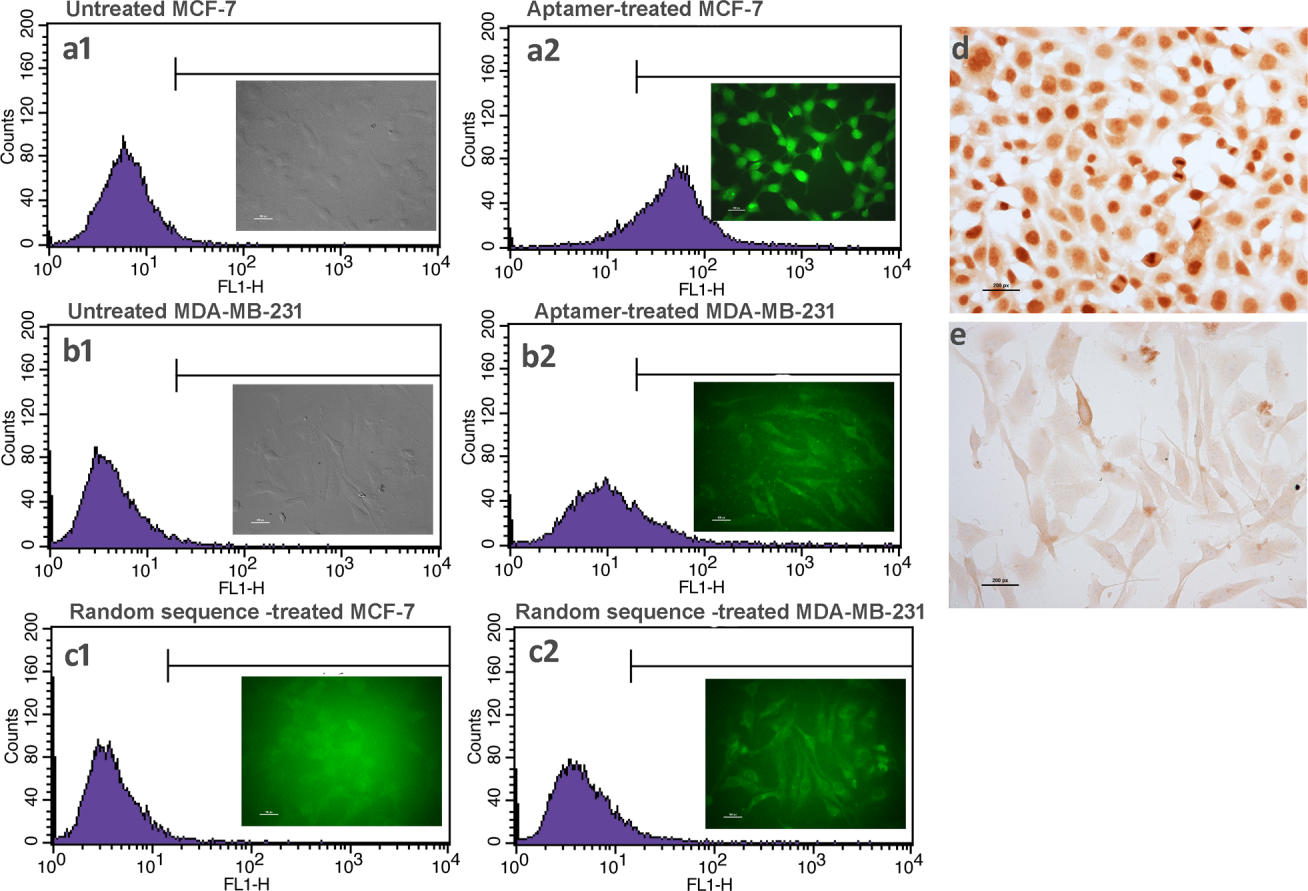
Specificity analysis of ERaptD4 using flow cytometry, fluorescent microscopy and cytochemistry. (**a1-a2**) Treatment of MCF-7 cells with fluorescent-tagged ERaptD4 produced a shift of 84% relative to unstained MCF-7 cells. Figure in inset provides the fluorescent microscopy image of the FAM-ERaptD4 treated cells. (**b1-b2**) A shift of 20% is observed in MDA-MB-231 cell upon treatment with fluorescent-labelled ERaptD4. (**c1-c2**) The treatment of MCF-7 cells with a random sequence (FAM-labelled non-enriched aptamer library) produced no shift, indicating the lack of binding by the random sequence. FAM-labelling of the non-enriched aptamer library is carried out using PCR amplification with FAM-forward primer and biotin-reverse primer. Strand separation is performed on streptavidin magnetic beads. (**d-e**) Aptacytochemistry of ERα-positive MCF-7 and ERα-deficient MDA-MB-231 cells. Size-bar on images a-c is 100 pixels (imaging at 63X). Size-bar on images d-e is 200 pixels (imaging at 20X).

Further confirmation of the target selectivity of ERaptD4 was made using flow cytometry and fluorescent microscopy assays. Flow cytometry was performed on aptamer (100 nM FAM-ERaptD4) treated MCF-7 and MDA-MB-231 cells (~2 ×10^5^) which were treated previously with 1% sodium azide to prevent the endocytic internalization of aptamers. As shown in Fig 4A1 and 4A2, the observed shift of 84% in aptamer treated MCF-7 cells in contrast to mere 20% shift for ERα-deficient MDA-MB-231 (Fig 4B1 and 4B2) evident the selective targeting of ERα by the ERaptD4 aptamer. The weak residual binding in the ER-deficient MDA-MB-231 cells could have resulted from non-selective internalization or cell adherence. Also, the lack of binding by the random DNA sequence (non-enriched aptamer library) confirmed the selective and high affinity binding of aptamer to its target (Fig 4C1 and 4C2). These observations were supported by chromogenic immunocytochemistry of ERα using ERaptD4. As shown in Fig 4D and 4E, the target-specific staining of the nuclei of ERα-positive MCF-7, which is comparably non-specific and less intense in the ERα-deficient MDA-MB-231 cells, suggest the specific binding of ERaptD4 to its target protein in ERα-positive breast cancer cells. Interestingly, in the obtained images, if we consider the intensity of specific staining as a measure to aptamer selectivity and background as an indicator to cross-reactivity, it is evident from the images that ERaptD4 specifically targets the ERα without cross reacting to other intracellular or extracellular components in the studied cell preparations.

### Aptamer- assisted detection of the ERα expression in breast cancer tissue sections

**Fig 5:**
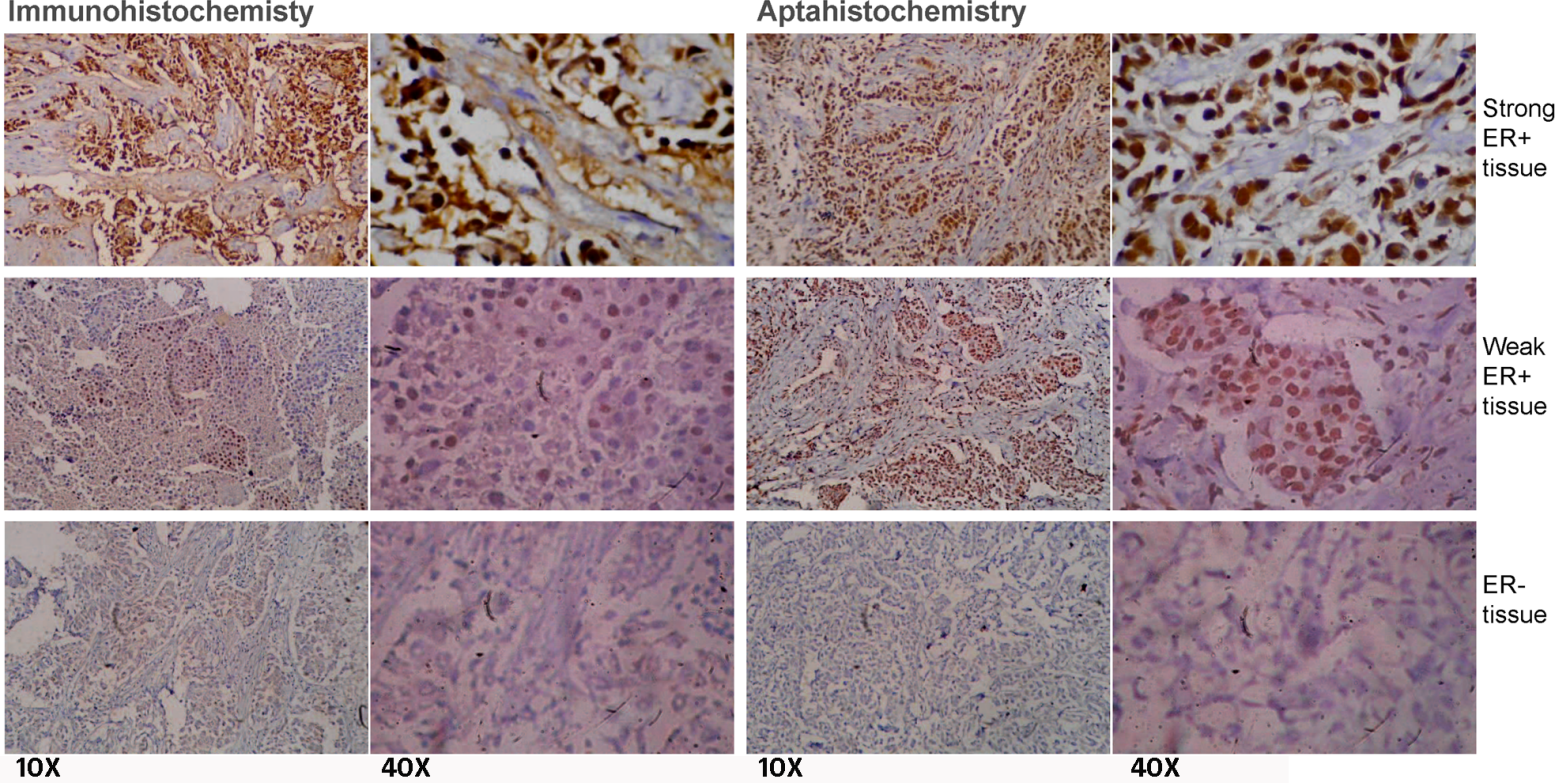
Staining of ERα-positive cells in breast cancer tissue samples. A comparative analysis of the aptahistochemistry and immunohistochemistry methods is made by analyzing the expression of ERα in breast tissue sections with ERα-antibody (sc-543) and ERaptD4 aptamer. The 10 X and 40 X represents the two magnifications of same images stained using antibody or aptamer.

Motivated by the observations suggesting an efficient staining of nuclear ERα in the cultured ERα-positive breast cancer cells by the ERaptD4, we attempted to ascertain the clinical applicability of this aptamer in evaluating the ERα expression, by selective staining of ERα-positive cells, to assist in breast cancer diagnosis and grading. For this, an aptamer-assisted histochemistry (aptahistochemistry) assay was performed using biotinylated-ERaptD4 on deparaffinised and hydrated tissue sections of breast cancer patients. The antigen was retrieved by two cycles of microwave heating and the non-specific sites and endogenous peroxidase activity were pre-blocked. As depicted in Fig 5, the ERaptD4 showed specific staining in the nuclei of ERα-positive breast cancer cells, where it stained the malignant duct cancer cells without any cross reactivity to fibroblasts, adipocytes, inflammatory cells or the extracellular components. Low expression of ERα (in moderate/weak ER+ve samples) could be seen as both decreased intensity of stain and lesser number of stained cells. For comparative analyses and to verify these results, histopathology and immunohistochemistry of same tissues were also determined using ERα-antibody through an immunohistochemistry assay. The immunocytochemistry also produced a similar pattern of staining in the strong (ERα positive), medium and negative samples of the ERα. In total, we have analyzed 50 samples of breast cancer patients. The results showed a strong agreement between immunohistochemistry and aptahistochemistry (kappa value = 0.930, p-value < 0.05 for strong ER+ve and the ER-ve samples, n = 30; kappa value = 0.823, p-value < 0.05 for the weak/moderate ER+ve samples, n = 20). Conclusively, the uniformity in the staining and the ease of detection was appreciable in aptamer stained tissues; this suggests that the selected ERaptD4 aptamer could be an efficient alternate to ERα-antibodies in clinical applications, such as histochemical examination of ERα in breast cancer samples.

## Discussion

ERα is a well characterized biomarker in breast cancer [7, 43, 44]. Ligands that bind to ERα are often utilized as probes for diagnostic and therapeutic purposes [8, 45]. Especially, the ER-antibodies have a long history of application in immunohistochemistry and other immunoassays for qualitative and quantitative detection of this receptor protein in breast cancer samples [12, 13]. However, the problem of cross-reactivity, loss of activity due to denaturation and the cost of diagnosis associated with antibodies have fuelled the search for new affinity ligands that can substitute conventional molecules in diagnostic applications [46]. As an alternate to conventional affinity ligands, the aptamers have been raised against a population of targets for application in diagnostics, therapeutics and targeted-drug delivery [26–30]. Aptamers are selected and synthesized in the *in vitro* systems with no reliance on living hosts. Additionally, the relatively low cost of production and low batch-to-batch variation allow the aptamers to fit in with clinical applications.

This study identified a DNA aptamer (ERaptD4) that targets the ERα with an affinity and specificity akin to that of ERα-antibodies. As the final enriched pool contains a large number of unique aptamer sequences, it is practically difficult to identify all sequences from this pool using traditional cloning and sequencing methods due to sampling of limited portion of the sequence space [47]. We overcame this limitation through high throughput sequencing to obtain the entire sequence space of the enriched pool. After selecting several probable aptamers from sequencing pool, based on affinity and specificity, we narrowed down the aptamer candidates for ERα to three, namely the ERaptD3, ERaptD4 and ERaptD8. In present study, we have only concentrated on ERaptD4, which was subsequently characterized for its potential application in the field of cancer biology, especially for detection of ERα in breast cancer samples. Binding affinity and the thermodynamic parameters for ERaptD4-ERα were determined through isothermal titration calorimetry. An association constant of 1.55±0.29×10^8^ M^-1^ and entropy (Δ*H*) and enthalpy (Δ*S*) values of 4.32×10^4^±801.1 cal/mol and -108 cal/mol/deg indeed suggested the favourable binding interactions of this complex. Interestingly, the stoichiometry (n) of 0.586 for ERaptD4-ERα binding hints the possibility of a dimer formation. Though we tried to validate this by cross linking the resulting ERaptD4-ERα complex and assessing its mobility on gel, we did not obtained any convincing results. Nevertheless, the uniqueness of the ERaptD4 aptamer, as determined through a combination of biochemical and immunological assays, include its ability to substitute ERα antibody in conventional assays such as enzyme linked immunosorbent assay (ELISA), western blotting, immunocytochemistry and immunohistochemistry. These characteristics of ERaptD4 aptamer can be harnessed for development of analytical methods and DNA-based biosensors for detection, purification or quantitation of ERα. Furthermore, unlike the previous aptamers of ERα, which targets the ERα-DBD [31], the ERaptD4 binds selectively and with considerable high affinity to the ERα-LBD. This may be useful for the *in vivo* applications of ERaptD4 as it would not disrupt replication or transcriptional processes of normal cells by competing with ligands that assembles at the DNA-binding domain of ERα. Further, the use of ERaptD4 in histochemistry can provide an efficient alternate to ERα-antibodies for qualitative and quantitative detection of this receptor protein in cancer samples.

## Supporting Information

S1 Fig Isothermal titration calorimetry for ERα-random library binding interaction. (DOCX)

S2 Fig Aptamer-assisted western blot. (DOCX)

S1 Table Summary of the SELEX screening. (DOCX)

S2 Table Copy number analysis of the sequences obtained from Illumina sequencing. (DOCX)

S3 Table Copy number of the top 50 sequences obtained from high throughput sequencing of the R9 pool of SELEX screening. (DOCX)

## References

[1] Fitzmaurice C, Dicker D, Pain A, Hamavid H, Moradi-Lakeh M, MacIntyre MF, et al The Global Burden of Cancer 2013. JAMA oncology. 2015;1(4):505–27. Epub 2015/07/17. 10.1001/jamaoncol.2015.0735 ; PubMed Central PMCID: PMCPmc4500822. 26181261 PMC4500822

[2] Society AAC. Cancer Facts & Figures 2015. 2015.

[3] Marino M, Galluzzo P, Ascenzi P. Estrogen signaling multiple pathways to impact gene transcription. Current genomics. 2006;7(8):497–508. Epub 2008/03/29. ; PubMed Central PMCID: PMCPmc2269003. 18369406 10.2174/138920206779315737 PMC2269003

[4] Moggs JG, Orphanides G. Estrogen receptors: orchestrators of pleiotropic cellular responses. EMBO reports. 2001;2(9):775–81. 10.1093/embo-reports/kve185 11559590 PMC1084040

[5] Driggers PH, Segars JH. Estrogen action and cytoplasmic signaling pathways. Part II: the role of growth factors and phosphorylation in estrogen signaling. Trends in endocrinology and metabolism: TEM. 2002;13(10):422–7. Epub 2002/11/15. ; PubMed Central PMCID: PMCPmc4152897. 12431838 10.1016/s1043-2760(02)00634-3 PMC4152897

[6] Fabris G, Marchetti E, Marzola A, Bagni A, Querzoli P, Nenci I. Pathophysiology of estrogen receptors in mammary tissue by monoclonal antibodies. Journal of steroid biochemistry. 1987;27(1–3):171–6. Epub 1987/01/01. . 3695478 10.1016/0022-4731(87)90307-4

[7] Viale G. The current state of breast cancer classification. Annals of Oncology. 2012;23(suppl 10):x207–x10. 10.1093/annonc/mds326 22987963

[8] Maximov PY, Lee TM, Jordan VC. The Discovery and Development of Selective Estrogen Receptor Modulators (SERMs) for Clinical Practice. Current Clinical Pharmacology. 2013;8(2):135–55. 10.2174/1574884711308020006 . 23062036 PMC3624793

[9] Lippman ME, Allegra JC. Quantitative estrogen receptor analyses: the response to endocrine and cytotoxic chemotherapy in human breast cancer and the disease-free interval. Cancer. 1980;46(12 Suppl):2829–34. Epub 1980/12/15. . 7448729 10.1002/1097-0142(19801215)46:12+<2829::aid-cncr2820461419>3.0.co;2-m

[10] Fisher B, Costantino JP, Wickerham DL, Redmond CK, Kavanah M, Cronin WM, et al Tamoxifen for prevention of breast cancer: report of the National Surgical Adjuvant Breast and Bowel Project P-1 Study. J Natl Cancer Inst. 1998;90(18):1371–88. Epub 1998/09/25. . 9747868 10.1093/jnci/90.18.1371

[11] Sawaki M, Wada M, Sato Y, Mizuno Y, Kobayashi H, Yokoi K, et al High-dose toremifene as first-line treatment of metastatic breast cancer resistant to adjuvant aromatase inhibitor: A multicenter phase II study. Oncol Lett. 2012;3(1):61–5. Epub 2012/06/29. 10.3892/ol.2011.449 ; PubMed Central PMCID: PMCPmc3362339. 22740857 PMC3362339

[12] Nicholson RI, Colin P, Francis AB, Keshra R, Finlay P, Williams M, et al Evaluation of an enzyme immunoassay for estrogen receptors in human breast cancers. Cancer research. 1986;46(8 Suppl):4299s–302s. Epub 1986/08/01. . 3524811

[13] Leclercq G, Bojar H, Goussard J, Nicholson RI, Pichon MF, Piffanelli A, et al Abbott monoclonal enzyme immunoassay measurement of estrogen receptors in human breast cancer: a European multicenter study. Cancer research. 1986;46(8 Suppl):4233s–6s. Epub 1986/08/01. . 2425942

[14] Shafie S, Brooks SC. Characteristics of the dextran-coated charcoal assay for estradiol receptor in breast cancer preparations. The Journal of laboratory and clinical medicine. 1979;94(5):784–98. Epub 1979/11/01. . 501205

[15] Goussard J, Lechevrel C, Martin PM, Roussel G. Comparison of monoclonal antibodies and tritiated ligands for estrogen receptor assays in 241 breast cancer cytosols. Cancer research. 1986;46(8 Suppl):4282s–7s. Epub 1986/08/01. . 2425945

[16] Remmele W, Schicketanz KH. Immunohistochemical determination of estrogen and progesterone receptor content in human breast cancer. Computer-assisted image analysis (QIC score) vs. subjective grading (IRS). Pathology, research and practice. 1993;189(8):862–6. Epub 1993/09/01. 10.1016/s0344-0338(11)81095-2 . 8302707

[17] Pertschuk LP, Kim DS, Nayer K, Feldman JG, Eisenberg KB, Carter AC, et al Immunocytochemical estrogen and progestin receptor assays in breast cancer with monoclonal antibodies. Histopathologic, demographic, and biochemical correlations and relationship to endocrine response and survival. Cancer. 1990;66(8):1663–70. Epub 1990/10/15. . 2208020 10.1002/1097-0142(19901015)66:8<1663::aid-cncr2820660802>3.0.co;2-c

[18] Kao J, Salari K, Bocanegra M, Choi YL, Girard L, Gandhi J, et al Molecular profiling of breast cancer cell lines defines relevant tumor models and provides a resource for cancer gene discovery. PloS one. 2009;4(7):e6146 Epub 2009/07/08. 10.1371/journal.pone.0006146 ; PubMed Central PMCID: PMCPmc2702084. 19582160 PMC2702084

[19] Goussard J. Paraffin section immunocytochemistry and cytosol-based ligand-binding assays for ER and PR detection in breast cancer: the time has come for more objectivity. Cancer letters. 1998;132(1–2):61–6. Epub 1999/07/09. . 10397454 10.1016/s0304-3835(98)00168-2

[20] Parise CA, Bauer KR, Brown MM, Caggiano V. Breast Cancer Subtypes as Defined by the Estrogen Receptor (ER), Progesterone Receptor (PR), and the Human Epidermal Growth Factor Receptor 2 (HER2) among Women with Invasive Breast Cancer in California, 1999–2004. The Breast Journal. 2009;15(6):593–602. 10.1111/j.1524-4741.2009.00822.x 19764994

[21] Lacroix M, Querton G, Hennebert P, Larsimont D, Leclercq G. Estrogen receptor analysis in primary breast tumors by ligand-binding assay, immunocytochemical assay, and northern blot: a comparison. Breast Cancer Res Treat. 2001;67(3):263–71. Epub 2001/09/20. . 11561772 10.1023/a:1017946810277

[22] Keefe AD, Cload ST. SELEX with modified nucleotides. Current Opinion in Chemical Biology. 2008;12(4):448–56. doi: 10.1016/j.cbpa.2008.06.028. 10.1016/j.cbpa.2008.06.028 18644461

[23] Bock LC, Griffin LC, Latham JA, Vermaas EH, Toole JJ. Selection of single-stranded DNA molecules that bind and inhibit human thrombin. Nature. 1992;355(6360):564–6. Epub 1992/02/06. 10.1038/355564a0 . 1741036

[24] Tuerk C, Gold L. Systematic evolution of ligands by exponential enrichment: RNA ligands to bacteriophage T4 DNA polymerase. Science (New York, NY). 1990;249(4968):505–10. Epub 1990/08/03. . 2200121 10.1126/science.2200121

[25] Robertson DL, Joyce GF. Selection in vitro of an RNA enzyme that specifically cleaves single-stranded DNA. Nature. 1990;344(6265):467–8. Epub 1990/03/29. 10.1038/344467a0 . 1690861

[26] Tombelli S, Minunni M, Mascini M. Analytical applications of aptamers. Biosensors and Bioelectronics. 2005;20(12):2424–34. doi: 10.1016/j.bios.2004.11.006. 15854817

[27] Song KM, Lee S, Ban C. Aptamers and their biological applications. Sensors (Basel, Switzerland). 2012;12(1):612–31. Epub 2012/03/01. 10.3390/s120100612 ; PubMed Central PMCID: PMCPmc3279232. 22368488 PMC3279232

[28] Germer K, Leonard M, Zhang X. RNA aptamers and their therapeutic and diagnostic applications. International Journal of Biochemistry and Molecular Biology. 2013;4(1):27–40. . 23638319 PMC3627066

[29] Ahirwar R, Nahar P. Development of an aptamer-affinity chromatography for efficient single step purification of Concanavalin A from Canavalia ensiformis. Journal of chromatography B, Analytical technologies in the biomedical and life sciences. 2015;997:105–9. Epub 2015/06/24. 10.1016/j.jchromb.2015.06.003 . 26102634

[30] Ahirwar R, Nahar P. Development of a label-free gold nanoparticle-based colorimetric aptasensor for detection of human estrogen receptor alpha. Anal Bioanal Chem. 2016;408(1):6 10.1007/s00216-015-9090-7 26476919

[31] He X, Chen J, Yie SM, Ye SR, Dong DD, Li K. Using a sequence of estrogen response elements as a DNA aptamer for estrogen receptors in vitro. Nucleic acid therapeutics. 2015;25(3):152–61. Epub 2015/03/04. 10.1089/nat.2014.0521 . 25734367

[32] Xu D, Chatakonda VK, Kourtidis A, Conklin DS, Shi H. In search of novel drug target sites on estrogen receptors using RNA aptamers. Nucleic acid therapeutics. 2014;24(3):226–38. Epub 2014/03/05. 10.1089/nat.2013.0474 ; PubMed Central PMCID: PMCPmc4026216. 24588102 PMC4026216

[33] Liu Z, Duan JH, Song YM, Ma J, Wang FD, Lu X, et al Novel HER2 aptamer selectively delivers cytotoxic drug to HER2-positive breast cancer cells in vitro. Journal of translational medicine. 2012;10:148 Epub 2012/07/24. 10.1186/1479-5876-10-148 ; PubMed Central PMCID: PMCPmc3583217. 22817844 PMC3583217

[34] Ahirwar R, Nahar S, Aggarwal S, Ramachandran S, Maiti S, Nahar P. In silico selection of an aptamer to estrogen receptor alpha using computational docking employing estrogen response elements as aptamer-alike molecules. Scientific reports. 2016;6:21285 10.1038/srep21285 Available: http://www.nature.com/articles/srep21285#supplementary-information. 26899418 PMC4761961

[35] Rio DC, Ares M Jr., Hannon GJ, Nilsen TW. Purification of RNA using TRIzol (TRI reagent). Cold Spring Harbor protocols. 2010;2010(6):pdb.prot5439. Epub 2010/06/03. 10.1101/pdb.prot5439 . 20516177

[36] Bora U, Chugh L, Nahar P. Covalent immobilization of proteins onto photoactivated polystyrene microtiter plates for enzyme-linked immunosorbent assay procedures. Journal of immunological methods. 2002;268(2):171–7. Epub 2002/09/07. . 12215385 10.1016/s0022-1759(02)00212-0

[37] Ahirwar R, Nahar P. Screening and identification of a DNA aptamer to concanavalin A and its application in food analysis. J Agric Food Chem. 2015;63(16):4104–11. Epub 2015/04/14. 10.1021/acs.jafc.5b00784 . 25865304

[38] Ahirwar R, Tanwar S, Parween S, Kumar A, Nahar P. Image-based detection of oligonucleotides—a low cost alternative to spectrophotometric or fluorometric methods. The Analyst. 2014;139(9):2186–92. Epub 2014/03/22. 10.1039/c3an02402d . 24653995

[39] Bolger AM, Lohse M, Usadel B. Trimmomatic: a flexible trimmer for Illumina sequence data. Bioinformatics (Oxford, England). 2014;30(15):2114–20. Epub 2014/04/04. 10.1093/bioinformatics/btu170 ; PubMed Central PMCID: PMCPmc4103590. 24695404 PMC4103590

[40] Cho M, Xiao Y, Nie J, Stewart R, Csordas AT, Oh SS, et al Quantitative selection of DNA aptamers through microfluidic selection and high-throughput sequencing. Proceedings of the National Academy of Sciences of the United States of America. 2010;107(35):15373–8. Epub 2010/08/14. 10.1073/pnas.1009331107 ; PubMed Central PMCID: PMCPmc2932614. 20705898 PMC2932614

[41] Tuominen VJ, Ruotoistenmaki S, Viitanen A, Jumppanen M, Isola J. ImmunoRatio: a publicly available web application for quantitative image analysis of estrogen receptor (ER), progesterone receptor (PR), and Ki-67. Breast cancer research: BCR. 2010;12(4):R56 Epub 2010/07/29. 10.1186/bcr2615 ; PubMed Central PMCID: PMCPmc2949645. 20663194 PMC2949645

[42] Kupakuwana GV, Crill JE, McPike MP, Borer PN. Acyclic Identification of Aptamers for Human alpha-Thrombin Using Over-Represented Libraries and Deep Sequencing. PloS one. 2011;6(5):e19395 10.1371/journal.pone.0019395 . 21625587 PMC3098231

[43] Banin Hirata BK, Oda JMM, Losi Guembarovski R, Ariza CB, Oliveira CECd, Watanabe MAE. Molecular Markers for Breast Cancer: Prediction on Tumor Behavior. Disease Markers. 2014;2014:12 10.1155/2014/513158 PMC3925609 24591761

[44] Weigel MT, Dowsett M. Current and emerging biomarkers in breast cancer: prognosis and prediction. Endocrine-related cancer. 2010;17(4):R245–R62. 10.1677/erc-10-0136 20647302

[45] Dhingra K. Antiestrogens—tamoxifen, SERMs and beyond. Investigational new drugs. 1999;17(3):285–311. Epub 2000/02/09. . 10665480 10.1023/a:1006348907994

[46] Marx V. Calling the next generation of affinity reagents. Nat Meth. 2013;10(9):829–33. 10.1038/nmeth.2607 23985727

[47] Lou X, Qian J, Xiao Y, Viel L, Gerdon AE, Lagally ET, et al Micromagnetic selection of aptamers in microfluidic channels. Proceedings of the National Academy of Sciences of the United States of America. 2009;106(9):2989–94. Epub 2009/02/10. 10.1073/pnas.0813135106 ; PubMed Central PMCID: PMCPmc2637280. 19202068 PMC2637280

