# Supplementary material for: Aptamer-Assisted Detection of the Altered Expression of Estrogen Receptor Alpha in Human Breast Cancer
Source: PLoS One. 2016 Apr 4;11(4):e0153001. doi: 10.1371/journal.pone.0153001 (PMC4820125; doi:10.1371/journal.pone.0153001)
Supplement: S1 Fig — (DOCX) [file pone.0153001.s001.docx]

**S1 Fig. ITC isotherms of ERα interactions with random DNA (non-enriched DNA library)**. For titration, the ERα concentration in 1.4 ml sample cell is taken as 1μM and random DNA concentration in the syringe was 10 μM. The top panel represents the raw heats of binding obtained upon titration of aptamer to ERα protein. The lower panel is the binding isotherm fitted to the raw data using one site model.
