# Supplementary material for: Aptamer-Assisted Detection of the Altered Expression of Estrogen Receptor Alpha in Human Breast Cancer
Source: PLoS One. 2016 Apr 4;11(4):e0153001. doi: 10.1371/journal.pone.0153001 (PMC4820125; doi:10.1371/journal.pone.0153001)
Supplement: S2 Fig — (DOCX) [file pone.0153001.s002.docx]

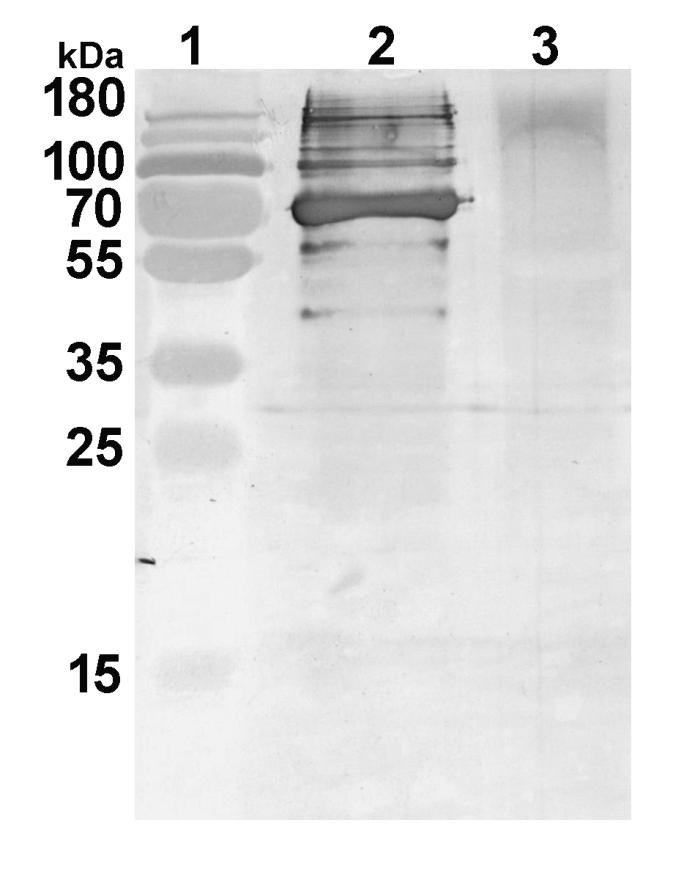


**S2 Fig. Aptamer-assisted western blot.** Target samples (Lane 2: full length ERα, MW=66 kDa; Lane 3: GST-tagged PR-LBD, MW= 56.4 kDa) are separated on 12% SDS PAGE and electroblotted to PVDF membrane. The immobilized proteins are detected by labelling them by complexing with primary detection molecule (1 µM ERaptD4) followed by horseradish peroxidase (HRP) conjugated streptavidin. The blot is developed using chromogenic substrate dye (OPD). Lane 1 represents the protein ladder (PageRuler™ Prestained Protein Ladder, 10-180 kDa).
