# Supplementary material for: Aptamer-Assisted Detection of the Altered Expression of Estrogen Receptor Alpha in Human Breast Cancer
Source: PLoS One. 2016 Apr 4;11(4):e0153001. doi: 10.1371/journal.pone.0153001 (PMC4820125; doi:10.1371/journal.pone.0153001)
Supplement: S1 Table — (DOCX) [file pone.0153001.s003.docx]

**S1 Table.** Summary of the SELEX screening.

| Screening rounds | Target protein (pmoles) | DNA library (nmoles)^†^ | Ratio of ERα to DNA library (moles) | Time of incubation (min) |
| --- | --- | --- | --- | --- |
| R1 | 100 | 50 | 1:500 | 120 |
| R2 | 100 | 1.0 | 1:10 | 60 |
| R3 | 75 | 1.0 | 1:13 | 60 |
| R4 | 50 | 1.0 | 1:20 | 30 |
| R5 | 25 | 1.0 | 1:40 | 30 |
| R6 | 10 | 1.0 | 1:100 | 15 |
| R7 | 5 | 1.0 | 1:200 | 15 |
| R8 | 5 | 1.0 | 1:200 | 15 |
| R9 | 5 | 1.0 | 1:200 | 15 |

^†^ First screening was performed with high amount of library to achieve maximum diversity.

^††^Subsequent screenings were performed with a ratio of protein to DNA that exerts a selection pressure on enrichment of superior binding sequences.
