# Supplementary material for: Aptamer-Assisted Detection of the Altered Expression of Estrogen Receptor Alpha in Human Breast Cancer
Source: PLoS One. 2016 Apr 4;11(4):e0153001. doi: 10.1371/journal.pone.0153001 (PMC4820125; doi:10.1371/journal.pone.0153001)
Supplement: S2 Table — (DOCX) [file pone.0153001.s004.docx]

**S2 Table. Copy number analysis of the sequences obtained from Illumina sequencing^†^**

| S. No. | Unique sequences (number) | Copy number |
| --- | --- | --- |
| 1 | 4 | ≥ 10000 |
| 2 | 4 | 1000- 9999 |
| 3 | 37 | 100- 999 |
| 4 | 2.8 × 10^4^ | 10- 99 |
| 5 | 0.3 × 10^6^ | 2-9 |
| 6 | 0.9 × 10^6^ | 1 |

^†^Total sequences (raw) in the enriched pool were 3.14 million.

After filtering, the sequences (unique sequences= 1.31 × 10^6^) remained to 3.03 × 10^6^
