# Supplementary material for: Aptamer-Assisted Detection of the Altered Expression of Estrogen Receptor Alpha in Human Breast Cancer
Source: PLoS One. 2016 Apr 4;11(4):e0153001. doi: 10.1371/journal.pone.0153001 (PMC4820125; doi:10.1371/journal.pone.0153001)
Supplement: S3 Table — (DOCX) [file pone.0153001.s005.docx]

**S3 Table.** Copy number of top 50 sequences obtained upon HTS of R9 of SELEX screening.

| S. No. | DNA sequence of the random region† | Copy number |
| --- | --- | --- |
| 1 | AGGTACAGAAGGCGCGACAGAAACTGCGGTCCCAGGGCGT | 17619 |
| 2 | GGCGGGCAGCGATTGTACCGGTACCACCTGGCAATGTAGA | 13762 |
| 3 | GGGGATAACGTCGTCACGTCGTGTCATCATTGGTTCAGTC | 11998 |
| 4 | CGTTGCATTTAGGTGCATTACGGGGGTTATCCGCTCTCTC | 11034 |
| 5 | GCAGCAGTGTCATATGAGGGCGTTCGTCAAATGTGCAGGG | 7263 |
| 6 | CGCATAGGCAAAACGGTGCGGTGCATATTCGTGACAAGCG | 2829 |
| 7 | GGACGGATGCACACACTACTGACCGGTTTTCGCCAGCCCC | 2628 |
| 8 | CGAGTAACGCTGTCTCTTCCGAATCGGGGGAAGGCGGAGG | 2406 |
| 9 | CACAGGGCTGTTTTTACGCAATGCTAGTGTTCGACTCAGT | 829 |
| 10 | CACTAATACTAGGCGGTCCACGCGCAGTTAAACCGGTCAA | 421 |
| 11 | CACATGCACGCTGTTACTCTTCAGACGGTTCGACTTGGCT | 249 |
| 12 | CGGTACAGAAGGCGCGACAGAAACTGCGGTCCCAGGGCGT | 244 |
| 13 | GGGTACAGAAGGCGCGACAGAAACTGCGGTCCCAGGGCGT | 223 |
| 14 | GCCCCAGCACATGACTCCAACGCCTAACCCCAACGACGGC | 193 |
| 15 | GGCGGGCAGCGATTGTACCGGGACCACCTGGCAATGTAGA | 187 |
| 16 | GCCACGAGAACATTCGCCTGCCCGTAAGATCGCTGCCGTC | 166 |
| 17 | ACTACAGTGAATGTGTTGCGAAAGTGCGTTATATTAACAT | 160 |
| 18 | GCACGAATAGGCAAGTGAATGTGGTTGCAACACCCGCCTC | 156 |
| 19 | GGGCCGGGGGTGGGGAACGTCCTCGTCTAGTGACATGTTG | 152 |
| 20 | GGGGAGTAGGCGCACCCACTATTCACAGGACAGCCCTGGT | 147 |
| 21 | GGACGGCGGGGCAGTGTACGTTCTGGTCAGCGGGTCGGGG | 147 |
| 22 | GGGGGGGGTTTATGGACTCTGTGGTTCTTTGATGTCTGTC | 143 |
| 23 | GGGCGGGAAGTTGCTCTCTGGTTGGTGTGCGTCGGGTGTC | 130 |
| 24 | GGGGGGCTGTGTAAACGGCCAACACACCCTACCACCCGTG | 127 |
| 25 | GGCAGGGACGATGGGTCGTTAGTTGCATTCTACGCGTGCC | 127 |
| 26 | GGCCGGGAATACATGAGACATAGACGAGCAACCCGTCGTG | 126 |
| 27 | GCACGGAGTAGAACACTGTATTAACATGCGGGTGCTGGTC | 125 |
| 28 | GAAGGAGCCGCACCCTATCCCTCGGTACGCTTCGTCCGTC | 121 |
| 29 | GGCGGGACAAGCGTTGGGATGTTTGTGAAAGGTGGTGGTC | 117 |
| 30 | GGCGGAGGAGGTGGTCATGGTCTGAGGGCACCCGTTGGTC | 117 |
| 31 | AGGGACAGAAGGCGCGACAGAAACTGCGGTCCCAGGGCGT | 117 |
| 32 | GGCGGGGGTACACGCAGAATGGGGGAAACCGTATCGCCGC | 115 |
| 33 | GGCAGCAGTAAGGGGGAATTCCCTACGTTCGTCACCCGCC | 115 |
| 34 | GGCGCGGGTATAGTGGGAACTCGAGTGCAATCACACTCTC | 113 |
| 35 | GCAACGAATCGCACATAGCTGACAGCCGCTACGTCCCCGC | 111 |
| 36 | GGCGGGTAAATGGCTGTTACGGGCTTCATTGTGTGTGTGC | 109 |
| 37 | GGGGTACAAGTTGACCCGCCGATAACCTAGTATCCGCGTG | 107 |
| 38 | GCACGGGAGCATAGCGACGGGCCACAGCTTCACTGCGGAC | 105 |
| 39 | GGCGGCAGAGGACGTGTCGGATGTCGCGAGTTTGACGTCC | 104 |
| 40 | GGCAGGACTCGGAACGGACACCGGCGTCAGGCTCCTCGTG | 103 |
| 41 | GGCACGAAACCTAGCCGATAGCACCTTAGTAGCCGTTGTG | 102 |
| 42 | GGCACATAGGAATTGACGGGTGGGGTAGTTGTTGTCCCGC | 102 |
| 43 | ACAAAGCGCGCTACAACCCTCTCGCTCCGTCTCCGCGTCC | 102 |
| 44 | GGCCGCACGAATAGGTCCTACGTGTCGAGGTGGATCTGTG | 100 |
| 45 | GGCACAGTTACGAAGCACAAAGTCCGGGTCGACCTGCCGC | 100 |
| 46 | GGGGGAACCGCCACGATAGTATCCACGAAATCGTGCCGTG | 97 |
| 47 | GGCGGCCGTCGAGATTGTCAGGGCGCTCGTCTCGCTCGGC | 95 |
| 48 | GCACGGTGAAGCGACTTGATAAGGAGAGGTAACTGTGCCC | 94 |
| 49 | GGAACGAGCCGGTGCGTCTGGACTATGATGCTTACGTGTC | 93 |
| 50 | GGGCGGGCTCGGGTAGTCGAACGTGGCTTCATGGGGTGGC | 92 |
